# The Effectiveness of a Low Glycemic Index/Load Diet on Cardiometabolic, Glucometabolic, and Anthropometric Indices in Children with Overweight or Obesity: A Systematic Review and Meta-Analysis

**DOI:** 10.3390/children10091481

**Published:** 2023-08-30

**Authors:** Ioustini Kalaitzopoulou, Xenophon Theodoridis, Evangelia Kotzakioulafi, Kleo Evripidou, Michail Chourdakis

**Affiliations:** 1 Laboratory of Hygiene, Social and Preventive Medicine and Medical Statistics, School of Medicine, Faculty of Health Sciences, Aristotle University of Thessaloniki, 54124 Thessaloniki, Greece; ioustinik@auth.gr (I.K.); xtheodoridis@auth.gr (X.T.); ekotzaki@auth.gr (E.K.); evripidou@auth.gr (K.E.); 2 Diabetes Center, 1st Propaedeutic Department of Internal Medicine, Medical School, “AHEPA” Hospital, Aristotle University of Thessaloniki, 54621 Thessaloniki, Greece

**Keywords:** systematic review, meta-analysis, low glycemic index, low glycemic load, obesity

## Abstract

(1) Background: This systematic review and meta-analysis aims to evaluate the impact of a low glycemic index (LGI) and low glycemic load (LGL) diet on children with overweight and obesity, analyzing any changes in anthropometric, cardiometabolic, and glucometabolic parameters. (2) Methods: Three electronic databases (PubMed, Scopus, CENTRAL), as well as clinical trial registries and reference lists of the included studies, were searched for eligible randomized control trials (RCTs). Two independent reviewers performed the screening of the studies, data extraction, and risk of bias assessment. Mean difference (MD) and 95% confidence intervals (CI) using a random effects model were calculated for each outcome. (3) Results: Eleven RCTs (n = 634) examining the effect of LGI diet versus control were identified. The synthesized data provided from the RCTs indicate no difference between intervention and control groups regarding primary outcomes (body weight (MD: −0.14; 95% CI −1.93 to 1.64, 5 trials), body mass index (BMI) (MD: −0.31; 95% CI −0.85 to 0.23, 6 trials), BMI z-score (MD: −0.03; 95% CI −0.09 to 0.02, 5 trials), and waist circumference (MD: −0.52; 95% CI −2.35 to 1.31, 5 trials)) and other measures of cardiometabolic and glucometabolic parameters. The majority of trials were classified as “some concerns”. (4) Conclusions: LGI and LGL diets do not seem to be associated with changes in adiposity, cardiometabolic or glucometabolic markers in children with overweight or obesity. Further research comparing the LGI diet to a high glycemic index diet, with proper methodological standards, is required to clarify the benefits of a LGI diet in this population.

## 1. Introduction

Childhood obesity has transmuted into a threatening public health issue. Persistent excess weight is mostly linked to multiple medical conditions, including non-alcoholic fatty liver disease (NAFLD), type 2 diabetes, as well as cardiovascular disease (CVD), and orthopedic complications [1]. The severity of the above-mentioned comorbidities commonly increases with the severity of obesity [1]. Additionally, obesity prevalence in childhood has a strong correlation with obesity in adulthood, especially in people who have a previous family history of obesity [2,3].

Poor eating habits and lack of physical activity are the main contributors to overweight or obesity, resulting in greater adolescent mortality, chronic diseases, and to the financial burden of healthcare [4,5]. Few interventions have demonstrated long-term effectiveness in reversing obesity, and even fewer have been widely adopted with a substantial positive public health impact [6]. The evidence suggests that among the most effective methods used to combat pediatric obesity are lifestyle interventions, particularly the combination of increased physical activity and the adoption of a healthier diet [7,8].

In terms of nutrition, many studies have focused on examining various combinations of dietary interventions (i.e., family-based, school-based, nutritional counseling, meal replacement, and diets with modified macronutrients) that may affect the prevention or treatment of childhood obesity [9,10]. Furthermore, published studies include diets that adjust the macronutrient ratio or modify the glycemic index (GI) or glycemic load (GL) [11]. The relationship between the GI and numerous diabetes-related parameters in young subjects has been adequately studied [9,10,12]. On the contrary, the relationship between GI and its effects on the obese pediatric population has received significantly less attention. The last comprehensive systematic review and meta-analysis (SR-MA) that analyzed the influence of low glycemic index (LGI) or low glycemic load (LGL) versus high GI/GL on anthropometric parameters, lipid profile, and glucose metabolism markers in children was published in 2015 [13]. As new studies on this issue have been published since then, we intended to conduct a new systematic review and meta-analysis to update current knowledge.

## 2. Materials and Methods

During the period spanning from September 2022 to June 2023, our endeavors were dedicated to the execution of a comprehensive SR-MA. This SR-MA was structured based on the Preferred Reporting Items for Systematic Reviews and Meta-Analyses (PRISMA) statement [14] (Supplementary Table S1). The protocol was pre-registered in the International Prospective Register of Systematic Reviews PROSPERO (CRD42022335409).

The PICO strategy was utilized to shape the research question and define the eligibility criteria. The applied PICO framework in the present systematic review is presented in Table 1.

### 2.1. Inclusion Criteria

We included only randomized controlled trials (RCTs) in which children with overweight or obesity up to 18 years old were enrolled, comparing LGI or LGL diets to any other intervention lasting no less than four weeks, and reporting any of our outcomes of interest. The definition of obesity was based on either WHO growth charts [15], CDC growth charts [16], International Obesity Task Force cut-offs [17], or as defined by any official national growth chart.

### 2.2. Exclusion Criteria

Studies that were not written in English, included children without obesity, did not meet our obesity diagnostic criteria, or were executed with a different study design, such as cluster randomized clinical trials, were excluded.

### 2.3. Search Strategy

Supplementary Table S2 shows the search strategy [18]. A thorough search method was created for PubMed and accordingly adapted for other databases. We searched PubMed^®^, Scopus^®^, as well as the Cochrane Central Register of Controlled Trials (CENTRAL), from inception to January 2023. Manual searches of the reference lists of the included trials were added to these searches as a complement. A clinical trial registry (clinicaltrials.gov) was also screened for upcoming, ongoing, or completed but unpublished studies. A manual search was carried out to locate articles that are not available in databases. Only English-language articles were included in our study.

### 2.4. Study Selection

Two independent examiners carried out the screening procedure. The obtained studies were imported into the Rayyan platform [19], where we semi-automatically removed duplicate records. Before assessing the full-text publications, each pair of reviewers examined the retrieved articles based on their titles and abstracts. Any disagreements were settled through discussion and, if required, by the senior author.

### 2.5. Data Extraction

Two researchers independently reviewed each included study’s relevant data and extracted it using a standardized form. This data included study design, study duration, country, participant characteristics, sample size, participant’s age, criteria for defining obesity, intervention and control types, dietary macronutrients, GI dose or GL, follow-up, funding sources, and outcome data. We computed GL from GIxCarbohydrate (g/d)/100 when GL was not supplied but GI and carbohydrates (g/d) were reported [20]. When available, we used total kilojoules to determine grams of carbohydrates per day when the amount of carbohydrates was stated as a proportion of energy. If not, we assumed a diet of 8368 kJ (2000 kcal). For missing data, the corresponding authors were contacted. Consensus was reached to settle potential differences.

### 2.6. Risk of Bias Assessment 

The Revised Cochrane Risk-of-Bias tool for randomized trials was used to assess the quality of the eligible studies (RoB 2.0) [21]. The overall quality of the included studies was deemed “low” if all domains are judged to be of low risk, or “high” if at least one domain is judged to be at high risk or at least three domains are judged to raise some concerns. The quality of a study is considered “some concerns” if at least one domain, but no more than two, is judged to raise some concerns, but there is no high risk of bias in any domain.

### 2.7. Data Analysis

We employed a random effects model to compute the pooled results for our outcomes. Mean difference (MD) and their corresponding 95% confidence intervals (CI) were calculated for each outcome. Heterogeneity was assessed using the τ^2^ and I^2^ index. The implementation of the prediction interval (PI) in the forest plots was interpreted as suggested by Higgins et al. [22]. The restricted estimated maximum likelihood (REML) estimator was used to quantify heterogeneity [23]. I^2^ values greater than 50% were deemed high heterogeneity [24]. In case of adequate number of studies, publications’ bias was assessed visually with funnel plots and formally tested with Egger’s test [25]. For the Egger’s test, *p*-values lower than 0.1 were considered the presence of publication bias. All analyses were performed with R software (version 4.2.1) using the meta package.

The order of preference of summary statistics were within-group mean differences and their respective variance measures. If mean differences were not provided, post-intervention data and their accounted standard deviation were extracted. If the standard deviation of the mean or MD was missing, the appropriate methods based on the Cochrane Handbook for systematic reviews of Interventions were used [24].

### 2.8. Quality of Evidence

The grading of the Recommendations Assessment, Development, and Evaluation (GRADE) tool was utilized to assess the certainty of the evidence [26]. Evidence was graded as “very high”, “high”, “low”, and “very low” quality.

## 3. Results

**Figure 1:**
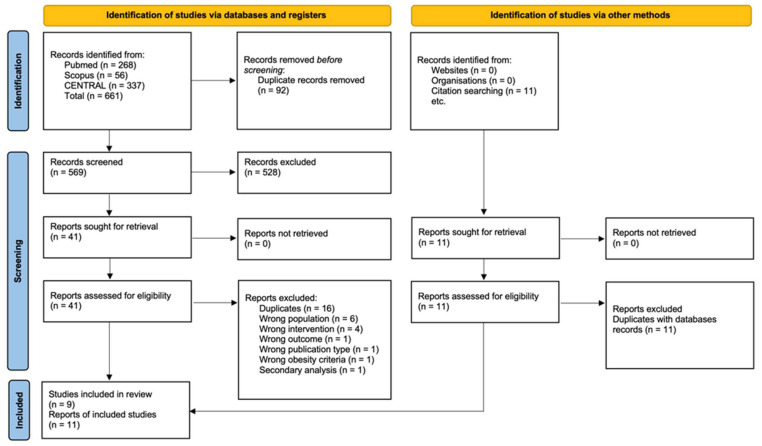
Flow diagram of the eligibility process.

Figure 1 shows the literature search and selection process in our study. Of the 661 total reports that were identified, 92 reports were detected through the Rayyan platform as duplicates, and 569 were excluded based on titles and abstracts. The remaining 41 reports were reviewed through the full text, and 30 were excluded based on eligibility criteria. The reasons for exclusion following a full-text assessment are listed in Supplementary Table S3. One of the included studies was multiply reported in three separate publications presenting distinct outcome data. A total of 11 reports (nine primary RCTs), involving 634 children who met obesity diagnostic criteria, were included in the final analysis.

### 3.1. Characteristics of the Included Studies

The characteristics of the nine included studies are summarized in Table 2 and Table 3. The median follow-up period for trials was 31.0 weeks (range 10.0–260.0 weeks), and males and females were distributed roughly equally in most studies (average percentage of total male participants 53%, range 0–82%). Participants’ age ranged from 7 to 18 years. All trials were performed in outpatient settings, with the majority conducted in the United States (37%), Italy, China, Iran, and Thailand. Only one of the trials used a multicenter approach [27].

The post-intervention GI and GL values in the LGI diet groups ranged from >50–75 (median 56) and 72–183.0 (median 87), respectively, and in the control groups, the GI values ranged from 52–90 (median 60) and the GL values from 83–255 (median 95). The macronutrient composition of both experimental and control diets differed among trials. Additionally, the amount of energy consumed by the participants appears to vary amongst the studies, and in some trials, one or both groups were exposed to caloric restriction.

### 3.2. Primary Outcomes

**Figure 2:**
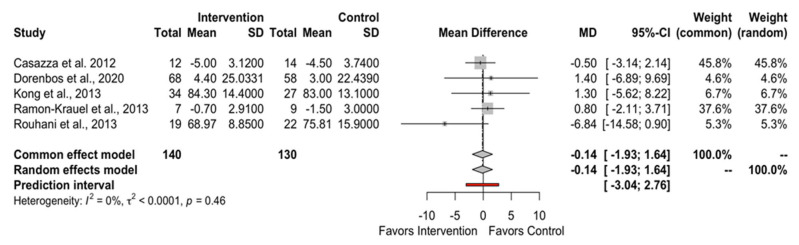
The overall effect of LGI/LGL diet on BW in childhood obesity [27,28,31,33,34].

**Figure 3:**
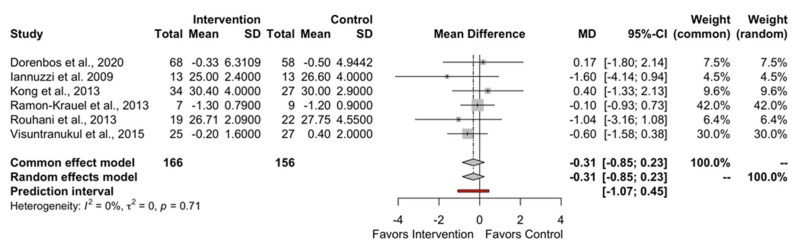
The overall effect of LGI/LGL diet on BMI in childhood obesity [27,29,31,33,34,37].

Figure 2 and Figure 3, and Supplementary Figures S1 and S2, depict the effect of low GI/GL dietary patterns on the primary outcomes of BW (MD: −0.14; 95% CI −1.93 to 1.64; PI −3.04 to 2.76), BMI (MD: −0.31; 95% CI −0.85 to 0.23; PI −1.07 to 0.45), BMI z-score (MD: −0.03; 95% CI −0.09 to 0.02; PI −0.12 to 0.06), and waist circumference (WC) (MD: −0.52; 95% CI −2.35 to 1.31; PI −3.49 to 2.45). There was no difference between the low GI/GL diets and control diets in any of our primary outcomes.

### 3.3. Secondary Outcomes

#### 3.3.1. Adiposity Markers

Regarding the secondary outcomes, Supplementary Figures S3 and S4 demonstrate the effect of low GI/GL dietary patterns on fat mass (FM) (MD: −0.23; 95% CI −2.61 to 2.16) and fat percentage (F%) (MD: −0.19; 95% CI −1.07 to 1.26; PI −2.46 to 2.65). In 4 out of the 11 trials reporting these data, low GI/GL diet did not lead to any changes in adiposity markers.

#### 3.3.2. Cardiometabolic Markers

Supplementary Figures S5–S13 illustrate the effect of low GI/GL dietary patterns on fasting blood glucose (FBG) (MD: −1.31; 95% CI −2.88 to 0.26; PI −3.37 to 0.75), fasting insulin (Fins) (MD: −0.61; 95% CI −4.16 to 2.94; PI −10.03 to 8.81), HOMA-IR (MD: −0.40; 95% CI −1.08 to 0.28; PI −2.19 to 1.39), total cholesterol (TC) (MD: −0.43; 95% CI −6.33 to 5.48; PI −10.61 to 9.76), low density lipoprotein cholesterol (LDL-c) (MD: 0.25; 95% CI −4.96 to 5.46; PI −8.20 to 8.70), high density lipoprotein cholesterol (HDL-c) (MD: 1.22; 95% CI −1.28 to 3.72; PI −2.33 to 4.77], triglycerides (TG) (MD: −6.43; 95% CI −19.37 to 6.50; PI −40.57 to 27.70), systolic blood pressure (SBP) (MD:−0.65; 95% CI −3.14 to 1.84; PI −5.45 to 4.15), and diastolic blood pressure (DBP) (MD −0.66; 95% CI −2.56 to 1.23; PI −4.35 to 3.02).

### 3.4. Quality of the Included Studies

The Cochrane risk of bias assessment tool for the included studies is shown in Supplementary Figures S14 and S15. The overall risk for most of the trials raised some concerns, while three had a high risk of bias. The studies’ lower quality was due to the downgrade regarding biases arising from the randomization process (Domain 1) and biases caused by deviations from the intended intervention (Domain 2).

### 3.5. Grade Assessment

Supplementary Table S4 depicts a summary of the GRADE assessments for the effect of LGI diet on primary outcomes. The evidence was graded as low for BW, BMI, BMI z-score, and WC, due to downgrading mainly in the domains of risk of bias and imprecision.

## 4. Discussion

This systematic review and meta-analysis included 11 randomized controlled trials incorporating 634 children with overweight or obesity. It was demonstrated that consumption of an LGI or LGL diet did not result in any changes regarding weight management and other anthropometric, cardiometabolic, or glucometabolic parameters when compared to nutritional recommendations or various diets.

Despite the abundance of RCTs in children and the embracement of the LGI diet as a feasible intervention in obesity, few publications summarize the findings of these clinical trials. The latest published review, examining the implication of an LGI diet on children with overweight or obesity, was in 2015 by Schwingshackl et al. [13]. The findings of this review were consistent with our results for the majority of the outcomes, except for the HOMA-IR and TG concentration which, although a modest reduction, lacked significance in our analysis. We excluded two studies from the latter review, as one trial used a different age range [38], while the second trial included both obese and non-obese children [39]. We also added three new studies to our review that were published after 2015, which might have contributed to the different results in those two parameters [27,36,37].

The typical approach to treating childhood obesity is reducing BW and adiposity, while also striving to prevent a recurrence [40,41]. A great number of scientific reports support the LGI diet as a highly promising approach to controlling body weight and preventing obesity [42,43], with an increasing number of researchers examining the relevance of GI since Jenkins et al. developed the “Glycemic index” term [44]. Our review, though, revealed that the LGI diet did not affect BW, BMI, WC, or adiposity markers in children and adolescents. Although research demonstrates that the LGI diet as an intervention appeared to be an effective treatment for adults with obesity in managing body composition aspects [45,46,47], the findings of the latest published review by Schwingshackl and colleagues examining the impact of the LGI diet on children with obesity are in line with our results [13]. Additionally, in two recent meta-analyses conducted by Zafar et al. and Perin et al., comparing the LGI diet to various control diets and the HGI diet, respectively, the LGI diet was not found to be superior to any of the control diets reviewed for weight loss in adults who were overweight [48,49]. Additionally, an important aspect is that BMI reduction can be achieved by reducing caloric intake, regardless of macronutrient composition, which might reflect our findings and the moderate influence of the LGI diet on weight control [50,51]. 

We additionally examined total cholesterol, LDL-c, HDL-c, triglycerides, SBP, and DBP, as prevention and elimination of cardiometabolic disease risk factors is a primary goal of obesity treatment [52,53]. Research proved that the consumption of LGI foods reduces total and LDL cholesterol concentrations in adults with obesity, reducing the risk of cardiometabolic disease [54,55]. Conversely, our study revealed that the LGI diet had little to no effect on cardiometabolic markers in the pediatric population. As the amount and type of fiber present can influence a food’s glycemic index [56], one proposed underlying mechanism behind the impact of the LGI diet on lipid profile is that dietary fiber limits bile acid and cholesterol re-absorption from the ileum, which may impair hepatic cholesterol synthesis [57,58]. However, the quantity of dietary fiber consumed was not documented. Hence, several studies included in our review reported an inadequate level of adherence to the intervention, which was interpreted as no alterations in pre- and post-measurements of glycemic load intake, which might influence the overall findings [27,31,32,37].

The LGI diet has been of particular interest to the population with obesity, as it was proven that it can slow down insulin secretion, attenuate the postprandial glucose response, and prolong satiety in non-diabetic subjects with obesity [48,59]. However, reducing the glycemic load intake was not related to lower fasting blood glucose or insulin levels compared to any control group in the present review. In accordance with our findings, the meta-analysis by Zafar and colleagues also revealed that LGI diets were not related to decreased fasting glucose levels when compared to HGI diets or any other control diet [49]. On the other hand, the LGI diet was described in an RCT as a low insulin response diet [60]. This study highlighted the amelioration of fasting plasma insulin and insulin resistance in children with hyperinsulinemia. However, not every child with obesity exhibits observed hyperinsulinemia and might not enhance the effect of an LGI diet in terms of reducing fasting plasma insulin [61].

The scientific community encourages the consumption of the LGI diet in individuals with overweight or obesity, as it has been shown to reduce the risk of type 2 diabetes [47]. Despite the fact that lowering GI has been cited as a viable strategy to control insulin sensitivity [62], the LGI diet in our study did not appear to have any impact on insulin sensitivity and HOMA-IR. The development of insulin resistance and increased adipose tissue deposition appear to be greatly impacted by the numerous metabolic changes that occur during puberty, which are triggered due to changes in the hormone profile [63,64]. Although insulin resistance decreases after puberty, the problem continues in children who were obese or become obese during adolescence [64,65].

Even though there have been clinical trials examining the impact of the LGI diet on children with obesity, comparisons with control groups have been evaluated using a wide spectrum of dietary treatments or nutritional counseling. On the other hand, the implications of consuming a HGI diet have been analyzed, highlighting the detrimental impacts on the general population and on those who have diabetes or obesity. For instance, the LGI diet appears to be superior to the HGI diet in terms of improving the lipid profile in the general population, patients with diabetes or obesity, thus reducing the overall risk of developing cardiometabolic syndrome [66,67,68]. However, research in the pediatric population is limited, particularly those comparing LGI versus HGI.

Finally, multiple studies that have examined the impact of LGI diet on metabolic markers are diverse among children and adult populations [13,48,49,69,70,71]. Most of these studies demonstrated modest or no changes in different metabolic components. A common limitation that these studies encounter is the highly questionable degree of compliance to the prescribed dietary interventions during the total duration of the study, but also by the level of restrictions [49]. Correspondingly, it has been reported that extremely strict interventions, like energy-restriction diets, would lead to a lower level of adherence. Children may struggle to adhere to a restrictive diet, resulting in a high dropout rate, as it has been observed that children tend to select forbidden foods when they are prohibited from consuming them [72]. Therefore, future clinical studies should focus on family-based lifestyle methods to enhance compliance, as they seemed to be superior to individual-level approaches [73].

### Strengths and Limitations

The strengths of our review include a thorough search and selection process that allowed comprehensive identification of all eligible studies and the use of intention to treat data for more conservative pooled estimates when those data were available [74].

Our study also had several limitations. Firstly, the studies included in our analysis might have a risk of bias. Secondly, different dietary patterns or healthy nutritional recommendations were used as control groups. Specifically, calorie intake was reduced in most of the trials in both intervention and control groups, which might have influenced the findings and acted as a confounding factor. The variability of the interventions, along with the small sample size of the included trials, might have affected the pooled effect estimate.

## 5. Conclusions

In conclusion, the LGI diet, compared with various control diets, seemed to have no benefit on body composition, cardiometabolic or glucometabolic profile in the pediatric population with overweight or obesity. It is not clear if these results are generalizable, due to the potential lack of adherence to the intervention. There is also a need for further research comparing the LGI diet to an HGI diet, with proper methodological standards, to clarify the health benefits of an LGI diet in this population.

## Figures and Tables

**Table 1:** Description of the PICO strategy.

PICO Acronym Criteria	PICO Items Relevant to Eligibility Criteria
(P) Population	Children with overweight or obesity as defined by WHO growth charts, CDC growth charts, IOTF cut-offs or any official national growth chart
(I) Intervention	LGI or LGL diet
(C) Comparator	Any dietary intervention
(O) Outcomes	BW, BMI, BMI z-score, WC, FM, F%, FBG, Fins, HOMA-IR, TC, LDL-c, HDL-c, TG, SBP, DBP

BMI: body mass index; BW: body weight; CDC: Centers for Disease Control and Prevention; DBP: diastolic blood pressure; F%: percentage of fat; FBG: fasting blood glucose; Fins: fasting insulin; FM: fat mass; HDL-c: high density lipoprotein cholesterol; HOMA-IR: homeostasis model assessment—insulin resistance; IOTF: International Obesity Task Force; LDL-c: low density lipoprotein cholesterol; LGI: low glycemic index; LGL: low glycemic load; SBP: systolic blood pressure; TC: total cholesterol; TG: triglycerides; WC: waist circumference; WHO: World Health Organization.

**Table 2:** Characteristics of the included randomized clinical trials.

First Author (Year) Country	Population	N	N Int.	N Con.	Sex (F/M)	Obesity Criteria	Participant’s Age (Years)	Baseline BMI	Baseline BMI
								Int. Group	Con. Group
								Mean (SD)	Mean (SD)
Casazza et al. [28] (2012) USA	Overweight/Obese AA girls	26	12	14	26/0	CDC Growth charts	9–14	NS	NS
Dorenbos et al. [27] (2021) Multicenter	Adolescents with overweight/obesity	126	68	58	74/52	IOTF cut-off points	10–18	30.1 (5.1)	29.3 (4.6)
Iannuzzi et al. [29] (2009) Italy	Obese children	26	13	13	14/12	CDC growth charts	7–13	28.3 (3.2)	28.4 (3.2)
Kirk et al. [30] (2012) USA	Obese children	102	35	36	59/43	CDC growth charts	7–12	29.2 (3.8)	29.1 (3.8)
Kong et al. [31] (2014) China	Obese adolescents	104	52	52	59/45	Hong Kong Growth Survey 1993	15–18	31.6 (4.2)	30.2 (3.5)
Mirza et al. [32] (2013) USA	Obese Hispanic children	113	57	56	55/58	CDC growth charts	7–14	31.1 (6.4)	30.03 (4.5)
Ramon-Krauel et. al. [33] (2013) USA	Obese children with fatty liver	17	8	9	3/14	CDC growth charts	8–17	31.3 (5.4)	34.0 (6.1)
Rouhani et al. [34,35] (2013) Iran	Obese/Overweight adolescent girls with the same pubertal status	50	25	25	50/0	WHO Growth Charts (BMI for age-girls)	13–18	27.9 (2.8)	28.8 (5.1)
Rouhani et al. [36] (2016) Iran	Obese/Overweight adolescent girls with the same pubertal status	50	19	22	50/0	WHO Growth Charts (BMI for age-girls)	13–18	27.9 (2.8)	28.8 (5.1)
Visuntranukul et al. [37] (2015) Thailand	Obese children	70	25	27	17/35	IOTF cut-off points	9–16	34.2 (5.8)	33.1 (6.6)

AA: African American; BMI: body mass index; CDC: Centers for Disease Control and Prevention; Con: control; F: female; M: male; Int: intervention; IOTF: International Obesity Task Force; SD: standard deviation; WHO: World Health Organization; NS: not stated.

**Table 3:** Intervention characteristics of the included randomized clinical trials.

First Author, Year	Study Duration (Weeks)	Intervention Type	Energy Restriction Int./Con.	Control Type	Dropout Rate (%) Intervention Group	Dropout Rate (%) Control Group	Macronutrients Int. (Pr/CHO/Fat)	Macronutrients Con. (Pr/CHO/Fat)	GI ^3^ Int.	GI ^3^ Con.	GL ^3^ Int. (g/d)	GL ^3^ Con.
Casazza et al., 2012 [28]	16	SPEC: reduced CHO diet	No/No	STAN: standard CHO	NS ^4^	NS	18/42/40	18/55/27	57	89	129	255
Dorenbos et al., 2021 [27]	260	HP/LGI diet	No/No	Moderate protein diet	63	59	25/45/30	15/55/30	50	52	87	89
Iannuzzi et al., 2009 [29]	24	LGI diet	Yes/Yes	HGI diet	NS	NS	20–25/50–55/25–30	20–25/50–55/25–30	60	90	NS	NS
Kirk et al., 2012 [30]	48	RGL diet	Yes/Yes	PC	13	13	16/50/34	14–52–34	49	54	84	83
Kong et al., 2014 [31]	48	LGI diet	Yes/Yes	Dietary Advice	34	48	15–25/45–50/30–35	10–20/55–60/25–30	75	77	183	211
Mirza et al., 2013 [32]	96	LGL diet	Yes/Yes	LF diet	42	44	20–25/45–50/30–35	15–20/55–60/25–30	56	55	87	84
Ramon-Krauel et al., 2013 [33]	24	LGL diet	No/No	LF diet	12	0	20–25/40/35–40	20–25/55–60/20	55	60	72	100
Rouhani et al., 2013 [34]	10	LGI diet	Yes/Yes	Healthy nutritional recommendation	24	20	16–18/53–56/27–30	16–18/53–56/27–30	NS ^1^		NS	
Rouhani et al., 2013 [35]	10	LGI diet	Yes/Yes	Healthy nutritional recommendation	24	20	16–18/53–56/27–30	16–18/53–56/27–30	NS ^1^		NS	
Rouhani et al., 2016 [36]	10	LGI diet	Yes/Yes	Healthy nutritional recommendation	24	20	16–18/53–56/27–30	16–18/53–56/27–30	NS ^1^		NS	
Visunthranukul et al., 2015 [37]	24	LGI diet	Yes/Yes	Standard Counseling	29	23	15–20/50–55/30–35	20/55/25	NS ^2^		NS	

BMI: body mass index, CHO: carbohydrate, Con: control, GI: glycemic index, GL: glycemic load, HGI: high glycemic index, HP: high protein, Int: intervention, LF: low fat, LGI: low glycemic index, LGL: low glycemic load, NA: not applicable; NS: not stated, PC: portion control, Pr: protein, RGL: reduced glycemic load, SPEC: specialized diet (reduced carbohydrate), STAN: standard diet (standard carbohydrate); ^1^ low glycemic index diet defined as glycemic index less than 50; ^2^ the low-GI diets include foods with GI less than 55; ^3^ post-intervention data of average glycemic index consumed; ^4^ dropout rate was 18% of the total number of participants.

## Data Availability

Not applicable.

## Supplementary Materials

The following supporting information can be downloaded at: https://www.mdpi.com/article/10.3390/children10091481/s1, Table S1: Preferred Reporting Items for Systematic Reviews and Meta-Analyses (PRISMA); Table S2: Search strategy for identifying randomized controlled trials on PubMed; Table S3: Excluded studies with reasons; Table S4: GRADE Assessment; Figure S1: Forest plot of BMI z-score; Figure S2: Forest plot of Waist circumference; Figure S3: Forest plot of Fat Mass; Figure S4: Forest plot of Fat percentage; Figure S5: Forest plot of Fasting Blood Glucose; Figure S6: Forest plot of Fasting Plasma Insulin; Figure S7: Forest plot of HOMA-IR; Figure S8: Forest plot of Total Cholesterol; Figure S9: Forest plot of LDL-c; Figure S10: Forest plot of HDL-c; Figure S11: Forest plot of Triglycerides; Figure S12: Forest plot of Systolic Blood Pressure; Figure S13: Forest plot of Diastolic Blood Pressure; Figure S14: Risk of bias assessment presented as traffic lights; Figure S15: Risk of bias assessment presented as bar plots.
