# Supplementary material for: The Effectiveness of a Low Glycemic Index/Load Diet on Cardiometabolic, Glucometabolic, and Anthropometric Indices in Children with Overweight or Obesity: A Systematic Review and Meta-Analysis
Source: Children (Basel). 2023 Aug 30;10(9):1481. doi: 10.3390/children10091481 (PMC10528374; doi:10.3390/children10091481)

Contents

Supplementary Table S1. *Preferred Reporting Items for Systematic Reviews and Meta-Analyses (PRISMA)*

Supplementary Table S2. Search strategy for identifying randomized controlled trials on PubMed.

Supplementary Table S3. Excluded studies with reasons

Supplementary Table S4. GRADE Assessment

Supplementary Figure S1 Forest plot of BMI z-score

Supplementary Figure S2. Forest plot of waist circumference

Supplementary Figure S3. Forest plot of Fat Mass

Supplementary Figure S4. Forest plot of Fat percentage

Supplementary Figure S5. Forest plot of Fasting Blood Glucose.

Supplementary Figure S6. Forest plot of Fasting Plasma Insulin

Supplementary Figure S7. Forest plot of HOMA-IR

Supplementary Figure S8. Forest plot of Total Cholesterol

Supplementary Figure S9. Forest plot of LDL-c

Supplementary Figure S10. Forest plot of HDL-c

Supplementary Figure S11. Forest plot of Triglycerides

Supplementary Figure S12. Forest plot of Systolic Blood Pressure

Supplementary Figure S13. Forest plot of Diastolic Blood Pressure

Supplementary Figure S14. Risk of bias assessment presented as traffic

Supplementary Figure S15. Risk of bias assessment presented as bar

Supplementary Table S1. Preferred Reporting Items for Systematic Reviews and Meta-Analyses (PRISMA).

Section and Topic	Item #	Checklist item	Location where item is reported
**TITLE**			
Title	1	Identify the report as a systematic review.	Page 1
**ABSTRACT**			
Abstract	2	See the PRISMA 2020 for Abstracts checklist.	Page 1
**INTRODUCTION**			
Rationale	3	Describe the rationale for the review in the context of existing knowledge.	Page 1-2
Objectives	4	Provide an explicit statement of the objective(s) or question(s) the review addresses.	Page 2
**METHODS**			
Eligibility criteria	5	Specify the inclusion and exclusion criteria for the review and how studies were grouped for the syntheses.	Pages 2-3
Information sources	6	Specify all databases, registers, websites, organisations, reference lists and other sources searched or consulted to identify studies. Specify the date when each source was last searched or consulted.	Page 3
Search strategy	7	Present the full search strategies for all databases, registers and websites, including any filters and limits used.	Table S2
Selection process	8	Specify the methods used to decide whether a study met the inclusion criteria of the review, including how many reviewers screened each record and each report retrieved, whether they worked independently, and if applicable, details of automation tools used in the process.	Page 3
Data collection process	9	Specify the methods used to collect data from reports, including how many reviewers collected data from each report, whether they worked independently, any processes for obtaining or confirming data from study investigators, and if applicable, details of automation tools used in the process.	Page 3
Data items	10a	List and define all outcomes for which data were sought. Specify whether all results that were compatible with each outcome domain in each study were sought (e.g. for all measures, time points, analyses), and if not, the methods used to decide which results to collect.	NA
	10b	List and define all other variables for which data were sought (e.g. participant and intervention characteristics, funding sources). Describe any assumptions made about any missing or unclear information.	Page 3
Study risk of bias assessment	11	Specify the methods used to assess risk of bias in the included studies, including details of the tool(s) used, how many reviewers assessed each study and whether they worked independently, and if applicable, details of automation tools used in the process.	Page 3
Effect measures	12	Specify for each outcome the effect measure(s) (e.g. risk ratio, mean difference) used in the synthesis or presentation of results.	Page 3-4
Synthesis methods	13a	Describe the processes used to decide which studies were eligible for each synthesis (e.g. tabulating the study intervention characteristics and comparing against the planned groups for each synthesis (item #5)).	
	13b	Describe any methods required to prepare the data for presentation or synthesis, such as handling of missing summary statistics, or data conversions.	Page 7
	13c	Describe any methods used to tabulate or visually display results of individual studies and syntheses.	Page 3-4
	13d	Describe any methods used to synthesize results and provide a rationale for the choice(s). If meta-analysis was performed, describe the model(s), method(s) to identify the presence and extent of statistical heterogeneity, and software package(s) used.	Page 3-4

Section and Topic	Item #	Checklist item	Location where item is reported
	13e	Describe any methods used to explore possible causes of heterogeneity among study results (e.g. subgroup analysis, meta-regression).	Page 3-4
	13f	Describe any sensitivity analyses conducted to assess robustness of the synthesized results.	NA
Reporting bias assessment	14	Describe any methods used to assess risk of bias due to missing results in a synthesis (arising from reporting biases).	Page 3
Certainty assessment	15	Describe any methods used to assess certainty (or confidence) in the body of evidence for an outcome.	Page 4
**RESULTS**			
Study selection	16a	Describe the results of the search and selection process, from the number of records identified in the search to the number of studies included in the review, ideally using a flow diagram.	Page 5
	16b	Cite studies that might appear to meet the inclusion criteria, but which were excluded, and explain why they were excluded.	Table S3
Study characteristics	17	Cite each included study and present its characteristics.	Page 5-6
Risk of bias in studies	18	Present assessments of risk of bias for each included study.	Figure S14-15
Results of individual studies	19	For all outcomes, present, for each study: (a) summary statistics for each group (where appropriate) and (b) an effect estimate and its precision (e.g. confidence/credible interval), ideally using structured tables or plots.	NA
Results of syntheses	20a	For each synthesis, briefly summarise the characteristics and risk of bias among contributing studies.	Page 7 and Figures S1-S13
	20b	Present results of all statistical syntheses conducted. If meta-analysis was done, present for each the summary estimate and its precision (e.g. confidence/credible interval) and measures of statistical heterogeneity. If comparing groups, describe the direction of the effect.	Page 7 and Figures S1-S13
	20c	Present results of all investigations of possible causes of heterogeneity among study results.	NA
	20d	Present results of all sensitivity analyses conducted to assess the robustness of the synthesized results.	NA
Reporting biases	21	Present assessments of risk of bias due to missing results (arising from reporting biases) for each synthesis assessed.	Figures S14-15
Certainty of evidence	22	Present assessments of certainty (or confidence) in the body of evidence for each outcome assessed.	Table S4
**DISCUSSION**			
Discussion	23a	Provide a general interpretation of the results in the context of other evidence.	Page 8
	23b	Discuss any limitations of the evidence included in the review.	Page 10
	23c	Discuss any limitations of the review processes used.	NA
	23d	Discuss implications of the results for practice, policy, and future research.	Page 9-10

Section and Topic	Item #	Checklist item	Location where item is reported
**OTHER INFORMATION**			
Registration and protocol	24a	Provide registration information for the review, including register name and registration number, or state that the review was not registered.	Page 2
	24b	Indicate where the review protocol can be accessed, or state that a protocol was not prepared.	Page 2
	24c	Describe and explain any amendments to information provided at registration or in the protocol.	NA
Support	25	Describe sources of financial or non-financial support for the review, and the role of the funders or sponsors in the review.	Page 10
Competing interests	26	Declare any competing interests of review authors.	Page 10
Availability of data, code and other materials	27	Report which of the following are publicly available and where they can be found: template data collection forms; data extracted from included studies; data used for all analyses; analytic code; any other materials used in the review.	Page 10

Supplementary Table S2. Search strategy for identifying randomized controlled trials on PubMed.

No	Keyword	Results
**#1**	glycaemic load[Title/Abstract]	287
**#2**	glycemic load[MeSH Terms]	349
**#3**	glycemic load[Title/Abstract]	1087
**#4**	glycaemic index[Title/Abstract]	1020
**#5**	glycemic ind*[Title/Abstract]	3440
**#6**	glycemic index[MeSH Terms]	3617
**#7**	glycemic index[Title/Abstract]	2991
**#8**	#1 OR #2 OR #3 OR #4 OR #5 OR #6 OR #7	6363
**#9**	adolescent[Title/Abstract]	153069
**#10**	adolescent[MeSH Terms]	2194796
**#11**	infant[MeSH Terms]	1234515
**#12**	infant[Title/Abstract]	246281
**#13**	toddler[Title/Abstract]	5652
**#14**	toddler[MeSH Terms]	0
**#15**	toddler[MeSH Terms] - Schema: all	0
**#16**	children[MeSH Terms]	2112413
**#17**	children[Title/Abstract]	1218968
**#18**	child[MeSH Terms]	2112413
**#19**	child[Title/Abstract]	471180
**#20**	paediatric[Title/Abstract]	72195

**#21**	paediatric[MeSH Terms]	62750
**#22**	pediatric[Title/Abstract]	331413
**#23**	pediatric[MeSH Terms]	62750
**#24**	#9 OR #10 OR # 11OR #12 OR #13 OR #14 OR #15 OR #16 OR #17 OR #18 OR #19 OR #20 OR #21 OR #22 OR #23	4344428
**#25**	adipose tissue[Title/Abstract]	83895
**#26**	adipose tissue[MeSH Terms]	109137
**#27**	adipos*[Title/Abstract]	128989
**#28**	adiposity[Title/Abstract]	29559
**#29**	adiposity[MeSH Terms]	15026
**#30**	overweight[MeSH Terms]	262837
**#31**	overweight[Title/Abstract]	84848
**#32**	obes*[Title/Abstract]	370171
**#33**	obesity[Title/Abstract]	307351
**#34**	obesity[MeSH Terms]	251787
**#35**	#25 OR #26 OR #27 OR #28 OR #29 OR #30 OR #31 OR #32 OR #33 OR #34	556980
**#36**	trial[Title/Abstract]	737336
**#37**	clinical trials[Title/Abstract]	309812
**#38**	randomized[Title/Abstract]	637077
**#39**	controlled clinical trial[Title/Abstract]	18447

**#40**	controlled clinical trial[Publication Type]	673077
**#41**	randomized controlled trial[Publication Type]	583043
**#42**	randomized controlled trial[Title/Abstract]	104392
**#43**	#36 OR #37 OR #38 OR #39 OR 40 OR #41 OR #42	1537713
**#44**	#8 AND #24 AND #35 AND #43	141

Supplementary Table S3. Excluded studies with reasons.

AO	Study ID	Reason	References
1	Armeno et al., 2011	Wrong population	https://doi.org/10.1515/jpem.2011.291
2	Astrup et al., 2013	Wrong population	https://doi.org/10.1096/fasebj.27.1_supplement.249.8
3	Ball et al., 2003	Wrong intervention	https://doi.org/10.1542/peds.111.3.488
4	Dalskov et al., 2014	Wrong population	https://doi.org/10.1017/S0007114513003760
5	Davis et al., 2007	Wrong intervention	https://doi.org/10.1038/oby.2009.19
6	Joslowski et al., 2015	Wrong intervention	https://doi.org/10.1016/j.clnu.2014.01.015
7	Kirk et al., 2017	Wrong outcome	https://doi.org/10.1089/chi.2017.0020
8	Papadaki et al., 2010	Wrong population	https://doi.org/10.1542/peds.2009-3633
9	Parillo et al., 2012	Wrong population	https://doi.org/10.3275/7909
10	Philippou et al., 2009	Wrong population	https://doi.org/10.1038/oby.2008.533
11	Rouhani et al., 2013	Wrong publication type	https://doi.org/10.1016/j.nut.2013.02.004
12	Spieth et al., 2000	Wrong obesity criteria	https://doi.org/10.1001/archpedi.154.9.947
13	Utari et al., 2019	Wrong intervention	https://doi.org/10.13181/mji.v28i2.2496
14	Visunthranukul et al., 2021	Secondary analysis	https://doi.org/10.1038/s41390-021-01463-0

Supplementary Table S4. GRADE Assessment.

Summary of findings:

LGI compared to control diets for obesity treatment

Patient or population: Children with overweight or obesity

Setting:

Intervention: Low glycemic index (LGI) diet

Comparison: Control diets

Outcomes	Anticipated absolute effects* (95% CI)		Relative effect (95% CI)	№ of participants (studies)	Certainty of the evidence (GRADE)	Comments
	Risk with other diets	Risk with LGI				
Body weight		MD **0.14 kg lower** (1.93 lower to 1.64 higher)	-	270 (5 RCTs)	⨁⨁◯◯ Low	The evidence suggests that LGI diet does not reduce body weight.
BMI		MD **0.31 kg/m² lower** (0.85 lower to 0.23 higher)	-	322 (6 RCTs)	⨁⨁◯◯ Low	The evidence suggests that LGI diet does not reduce BMI.
BMI z-score		MD **0.03 lower** (0.09 lower to 0.02 higher)	-	374 (5 RCTs)	⨁⨁◯◯ Low	The evidence suggests that LGI diet does not reduce BMIz.
Waist Circumference (WC)		MD **0.52 cm lower** (2.35 lower to 1.31 higher)	-	196 (5 RCTs)	⨁⨁◯◯ Low	The evidence suggests that LGI diet does not reduce WC.

***The risk in the intervention group** (and its 95% confidence interval) is based on the assumed risk in the comparison group and the **relative effect** of the intervention (and its 95% CI).

CI: confidence interval; **MD:** mean difference

GRADE Working Group grades of evidence
High certainty: we are very confident that the true effect lies close to that of the estimate of the effect.
Moderate certainty: we are moderately confident in the effect estimate: the true effect is likely to be close to the estimate of the effect, but there is a possibility that it is substantially different.
Low certainty: our confidence in the effect estimate is limited: the true effect may be substantially different from the estimate of the effect.
Very low certainty: we have very little confidence in the effect estimate: the true effect is likely to be substantially different from the estimate of effect.

Supplementary Figure S1 Forest plot of BMI z-score

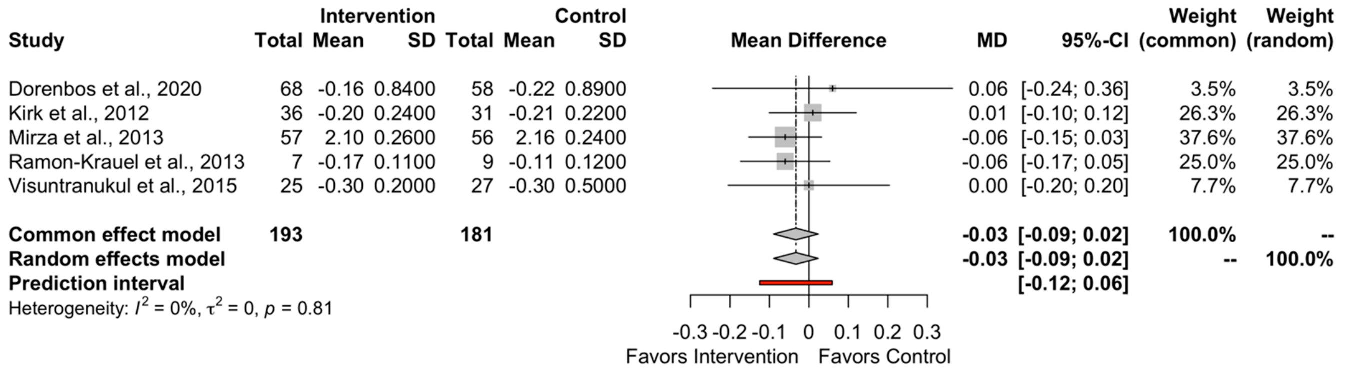

Supplementary Figure S2. Forest plot of Waist circumference

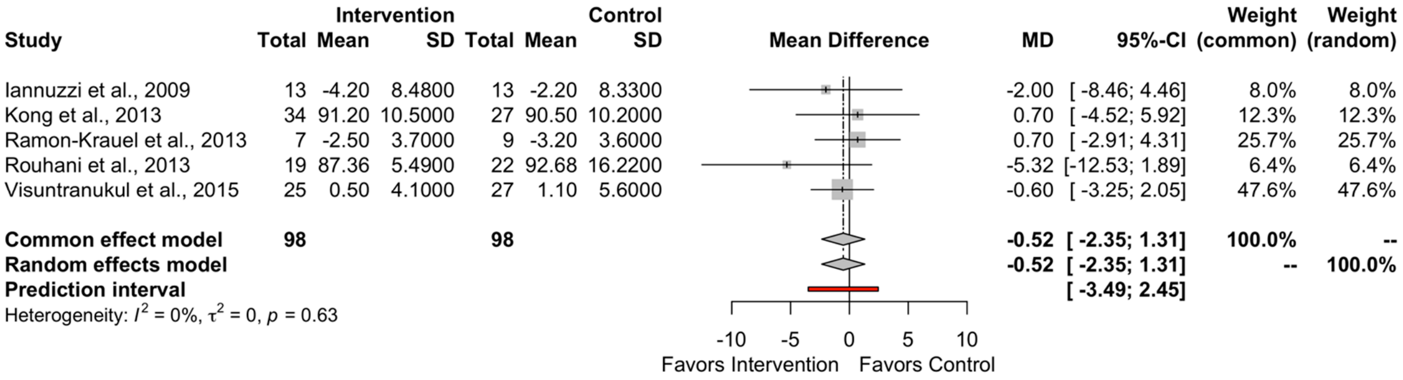

Supplementary Figure S3. Forest plot of Fat Mass.

Study	Intervention Total	Intervention Mean	Intervention SD	Control Total	Control Mean	Control SD	MD	95%-CI	Weight (common)	Weight (random)
Casazza et al., 2012	12	-3.40	3.4600	14	-2.90	3.3700	-0.50	[-3.14; 2.14]	81.7%	81.7%
Dorenbos et al., 2020	68	5.00	15.1461	58	4.00	16.5440	1.00	[-4.58; 6.58]	18.3%	18.3%
**Common effect model**	**80**			**72**			**-0.23**	**[-2.61; 2.16]**	**100.0%**	**--**
**Random effects model**							**-0.23**	**[-2.61; 2.16]**	**--**	**100.0%**

Heterogeneity: $I^2 = 0\%$, $\tau^2 = 0$, $p = 0.63$

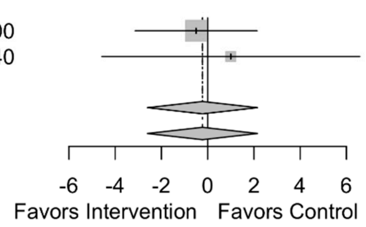

Supplementary Figure S4. Forest plot of Fat percentage.

Study	Intervention Total	Intervention Mean	Intervention SD	Control Total	Control Mean	Control SD	MD	95%-CI	Weight (common)	Weight (random)
Casazza et al., 2012	12	-3.40	3.1200	14	-3.20	2.6200	-0.20	[-2.44; 2.04]	27.1%	27.1%
Dorenbos et al., 2020	68	1.40	9.6800	58	2.00	10.6490	-0.60	[-4.18; 2.98]	10.6%	10.6%
Kong et al., 2013	34	38.50	8.9000	27	35.90	7.4000	2.60	[-1.49; 6.69]	8.1%	8.1%
Visuntranukul et al., 2015	25	0.10	3.0000	27	0.10	2.8000	0.00	[-1.58; 1.58]	54.2%	54.2%
**Common effect model**	**139**			**126**			**0.09**	**[-1.07; 1.26]**	**100.0%**	**--**
**Random effects model**							**0.09**	**[-1.07; 1.26]**	**--**	**100.0%**
**Prediction interval**								**[-2.46; 2.65]**		

Heterogeneity: $I^2 = 0\%$, $\tau^2 = 0$, $p = 0.64$

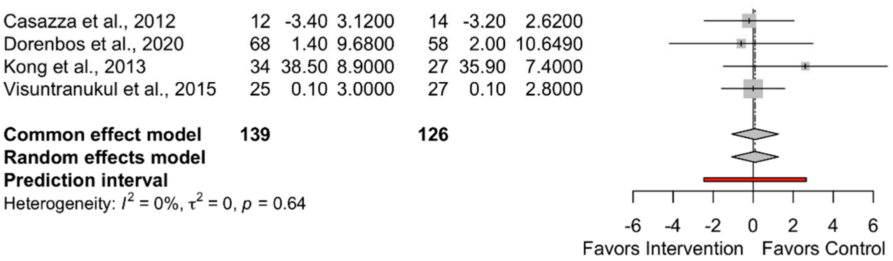

Supplementary Figure S5. Forest plot of Fasting Blood Glucose.

Study	Intervention Total	Intervention Mean	Intervention SD	Control Total	Control Mean	Control SD	MD	95%-CI	Weight (common)	Weight (random)
Dorenbos et al., 2020	68	5.40	18.9537	58	7.20	20.5564	-1.80	[-8.75; 5.15]	5.1%	5.1%
Iannuzzi et al., 2009	13	82.10	6.4000	13	85.10	8.5000	-3.00	[-8.78; 2.78]	7.4%	7.4%
Kirk et al., 2012	36	93.90	7.2000	31	93.60	5.5700	0.30	[-2.76; 3.36]	26.3%	26.3%
Kong et al., 2013	34	84.69	7.2000	27	84.69	9.0100	0.00	[-4.17; 4.17]	14.2%	14.2%
Ramon-Krauel et al., 2013	7	-0.50	5.5600	9	1.80	5.7000	-2.30	[-7.85; 3.25]	8.0%	8.0%
Rouhani et al., 2016	25	93.63	4.9000	25	94.99	6.7500	-1.36	[-4.63; 1.91]	23.1%	23.1%
Visuntranukul et al., 2015	27	-1.80	7.2100	25	1.80	7.2100	-3.60	[-7.52; 0.32]	16.0%	16.0%
**Common effect model**	**210**			**188**			**-1.31**	**[-2.88; 0.26]**	**100.0%**	**--**
**Random effects model**							**-1.31**	**[-2.88; 0.26]**	**--**	**100.0%**
**Prediction interval**								**[-3.37; 0.75]**		

Heterogeneity: $I^2 = 0\%$, $\tau^2 = 0$, $p = 0.78$

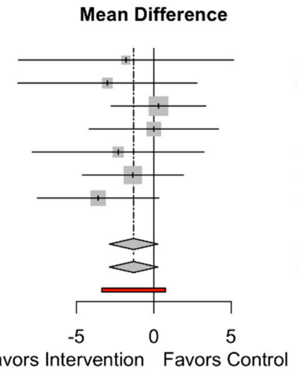

Supplementary Figure S6. Forest plot of Fasting Plasma Insulin.

Study	Intervention Total	Intervention Mean	Intervention SD	Control Total	Control Mean	Control SD	MD	95%-CI	Weight (common)	Weight (random)
Dorenbos et al., 2020	68	4.03	23.9182	58	1.90	21.1840	2.13	[-5.75; 10.01]	9.8%	13.5%
Iannuzzi et al., 2009	13	12.10	5.0000	13	15.10	7.9000	-3.00	[-8.08; 2.08]	23.5%	22.0%
Kirk et al., 2012	36	22.00	13.9200	31	22.80	10.8000	-0.80	[-6.73; 5.13]	17.2%	18.9%
Ramon-Krauel et al., 2013	7	-1.50	18.2600	9	-18.50	17.4000	17.00	[-0.67; 34.67]	1.9%	3.7%
Rouhani et al., 2016	25	18.03	7.1500	25	15.93	7.9500	2.10	[-2.09; 6.29]	34.5%	25.7%
Visuntranukul et al., 2015	25	-8.50	13.5000	27	-0.80	11.3000	-7.70	[-14.49; -0.91]	13.1%	16.2%
**Common effect model**	**174**			**163**			**-0.59**	**[-3.05; 1.87]**	**100.0%**	**--**
**Random effects model**							**-0.61**	**[-4.16; 2.94]**	**--**	**100.0%**
**Prediction interval**								**[-10.03; 8.81]**		

Heterogeneity: $I^2 = 54\%$, $\tau^2 = 8.2218$, $p = 0.05$

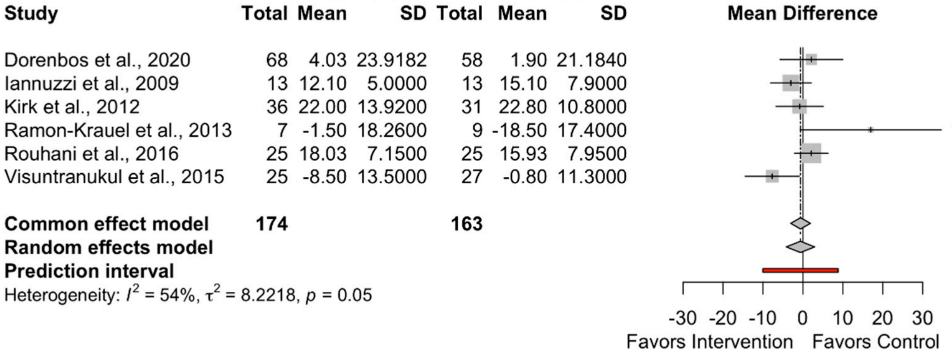

Supplementary Figure S7. Forest plot of HOMA-IR.

Study	Intervention Total	Intervention Mean	Intervention SD	Control Total	Control Mean	Control SD	MD	95%-CI	Weight (common)	Weight (random)
Dorenbos et al., 2020	68	0.85	3.6182	58	0.89	3.3849	-0.04	[-1.26; 1.18]	14.0%	17.5%
Iannuzzi et al., 2009	13	2.40	1.1000	13	3.20	1.8000	-0.80	[-1.95; 0.35]	15.9%	18.8%
Mirza et al., 2013	57	2.44	1.6600	56	3.12	2.1500	-0.68	[-1.39; 0.03]	41.6%	28.1%
Ramon-Krauel et al., 2013	7	-0.30	3.9600	9	-3.90	3.9000	3.60	[-0.29; 7.49]	1.4%	2.8%
Rouhani et al., 2016	25	4.20	1.7500	25	3.77	2.0000	0.43	[-0.61; 1.47]	19.3%	20.7%
Visuntranukul et al., 2015	25	-1.90	3.2000	27	0.10	2.8000	-2.00	[-3.64; -0.36]	7.8%	12.0%
**Common effect model**	**195**			**188**			**-0.44**	**[-0.90; 0.02]**	**100.0%**	**--**
**Random effects model**							**-0.40**	**[-1.08; 0.28]**	**--**	**100.0%**
**Prediction interval**								**[-2.19; 1.39]**		

Heterogeneity: $I^2 = 57\%$, $\tau^2 = 0.2949$, $p = 0.04$

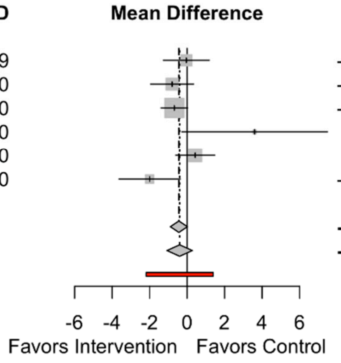

Supplementary Figure S8. Forest plot of Total Cholesterol.

Study	Intervention Total	Intervention Mean	Intervention SD	Control Total	Control Mean	Control SD	MD	95%-CI	Weight (common)	Weight (random)
Iannuzzi et al., 2009	13	157.50	29.9000	13	171.10	29.1000	-13.60	[-36.28; 9.08]	6.6%	6.7%
Kirk et al., 2012	36	153.00	24.0000	31	155.00	27.8400	-2.00	[-14.55; 10.55]	21.4%	21.5%
Kong et al., 2013	34	154.40	30.8900	27	162.16	30.8900	-7.76	[-23.37; 7.85]	13.8%	14.1%
Rouhani et al., 2013	25	4.77	17.0000	25	-0.30	15.9500	5.07	[-4.07; 14.21]	40.4%	39.6%
Visuntranukul et al., 2015	25	-3.86	27.0300	27	-3.86	23.1700	0.00	[-13.73; 13.73]	17.9%	18.1%
**Common effect model**	**133**			**123**			**-0.35**	**[-6.15; 5.46]**	**100.0%**	**--**
**Random effects model**							**-0.43**	**[-6.33; 5.48]**	**--**	**100.0%**
**Prediction interval**								**[-10.61; 9.76]**		

Heterogeneity: $I^2 = 0\%$, $\tau^2 = 1.1602$, $p = 0.46$

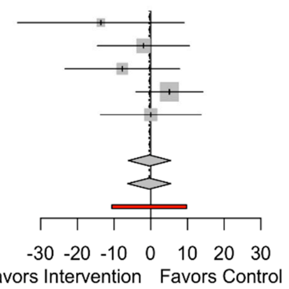

Supplementary Figure S9. Forest plot of LDL-c.

Study	Intervention Total	Intervention Mean	Intervention SD	Control Total	Control Mean	Control SD	MD	95%-CI	Weight (common)	Weight (random)
Kirk et al., 2012	36	87.50	20.4000	31	89.30	25.0500	-1.80	[-12.85; 9.25]	22.2%	22.2%
Kong et al., 2013	34	88.80	27.3000	27	92.66	27.3000	-3.86	[-17.65; 9.93]	14.2%	14.2%
Ramon-Krauel et al., 2013	7	6.10	25.6600	9	0.90	26.4000	5.20	[-20.47; 30.87]	4.1%	4.1%
Rouhani et al., 2013	25	3.47	17.1000	25	-2.07	14.6000	5.54	[-3.27; 14.35]	34.9%	34.9%
Visuntranukul et al., 2015	25	-3.86	19.3100	27	0.00	19.3100	-3.86	[-14.36; 6.64]	24.6%	24.6%
**Common effect model**	**127**			**119**			**0.25**	**[-4.96; 5.46]**	**100.0%**	**--**
**Random effects model**							**0.25**	**[-4.96; 5.46]**	**--**	**100.0%**
**Prediction interval**								**[-8.20; 8.70]**		

Heterogeneity: $I^2 = 0\%$, $\tau^2 = 0$, $p = 0.63$

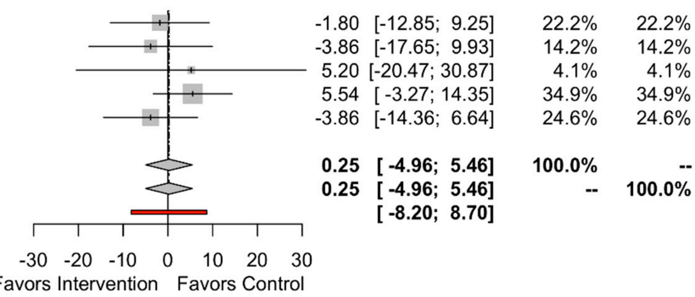

Supplementary Figure S10. Forest plot of HDL-c.

Study	Intervention Total	Intervention Mean	Intervention SD	Control Total	Control Mean	Control SD	MD	95%-CI	Weight (common)	Weight (random)
Iannuzzi et al., 2009	13	53.00	12.5000	13	51.00	5.9000	2.00	[-5.51; 9.51]	11.1%	11.1%
Kirk et al., 2012	36	49.20	9.0000	31	48.20	11.6900	1.00	[-4.06; 6.06]	24.5%	24.5%
Ramon-Krauel et al., 2013	7	4.20	8.4700	9	-1.70	8.7000	5.90	[-2.57; 14.37]	8.7%	8.7%
Rouhani et al., 2013	25	0.82	15.3000	25	-1.60	13.5000	2.42	[-5.58; 10.42]	9.8%	9.8%
Visuntranukul et al., 2015	25	0.00	11.5800	27	0.00	7.7200	0.00	[-5.39; 5.39]	21.6%	21.6%
Kong et al., 2014	34	46.33	7.7200	27	46.33	11.5800	0.00	[-5.08; 5.08]	24.3%	24.3%
**Common effect model**	**140**			**132**			**1.22**	**[-1.28; 3.72]**	**100.0%**	**--**
**Random effects model**							**1.22**	**[-1.28; 3.72]**	**--**	**100.0%**
**Prediction interval**								**[-2.33; 4.77]**		

Heterogeneity: $I^2 = 0\%$, $\tau^2 = 0$, $p = 0.89$

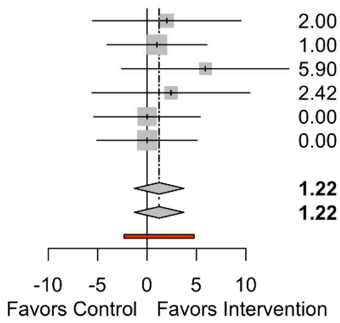

Supplementary Figure S11. Forest plot of Triglycerides.

Study	Intervention Total	Intervention Mean	Intervention SD	Control Total	Control Mean	Control SD	MD	95%-CI	Weight (common)	Weight (random)
Iannuzzi et al., 2009	13	67.10	16.6000	13	94.10	40.6000	-27.00	[-50.84; -3.16]	16.4%	17.0%
Kirk et al., 2012	36	81.00	42.0000	31	88.00	44.5400	-7.00	[-27.83; 13.83]	21.4%	19.7%
Kong et al., 2013	34	97.40	44.3000	27	115.05	44.3000	-17.65	[-40.03; 4.73]	18.6%	18.3%
Ramon-Krauel et al., 2013	7	11.20	25.1300	9	-10.60	39.9000	21.80	[-10.23; 53.83]	9.1%	11.6%
Rouhani et al., 2013	25	25.10	65.1000	25	7.36	46.3000	17.74	[-13.57; 49.05]	9.5%	12.0%
Visuntranukul et al., 2015	25	-8.85	35.4000	27	0.00	35.4000	-8.85	[-28.11; 10.41]	25.1%	21.3%
**Common effect model**	**140**			**132**			**-7.76**	**[-17.40; 1.89]**	**100.0%**	**--**
**Random effects model**							**-6.43**	**[-19.37; 6.50]**	**--**	**100.0%**
**Prediction interval**								**[-40.57; 27.70]**		

Heterogeneity: $I^2 = 45\%$, $\tau^2 = 107.6337$, $p = 0.11$

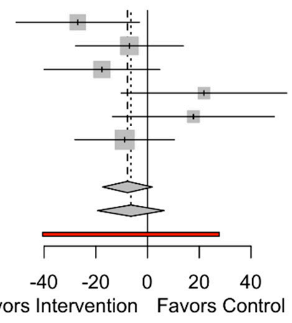

Supplementary Figure S12. Forest plot of Systolic Blood Pressure.

Study	Intervention Total	Intervention Mean	Intervention SD	Control Total	Control Mean	Control SD	MD	95%-CI	Weight (common)	Weight (random)
Iannuzzi et al., 2009	13	111.50	12.3000	13	108.50	9.0000	3.00	[-5.28; 11.28]	7.6%	8.4%
Kirk et al., 2012	36	100.00	10.2000	31	99.70	9.4700	0.30	[-4.41; 5.01]	23.4%	22.6%
Kong et al., 2013	34	123.00	10.0000	27	120.70	10.6000	2.30	[-2.92; 7.52]	19.1%	19.1%
Ramon-Krauel et al., 2013	7	2.90	7.9400	9	1.50	8.1000	1.40	[-6.51; 9.31]	8.3%	9.1%
Rouhani et al., 2013	19	115.26	9.0200	22	120.00	8.7200	-4.74	[-10.19; 0.71]	17.5%	17.7%
Visuntranukul et al., 2015	25	109.00	9.0000	27	112.00	8.0000	-3.00	[-7.64; 1.64]	24.1%	23.1%
**Common effect model**	**134**			**129**			**-0.70**	**[-2.98; 1.58]**	**100.0%**	**--**
**Random effects model**							**-0.65**	**[-3.14; 1.84]**	**--**	**100.0%**
**Prediction interval**								**[-5.45; 4.15]**		

Heterogeneity: $I^2 = 10\%$, $\tau^2 = 1.3713$, $p = 0.35$

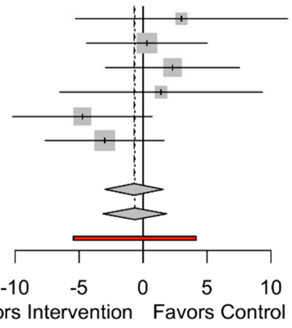

Supplementary Figure S13. Forest plot of Diastolic Blood Pressure.

Study	Intervention Total	Intervention Mean	Intervention SD	Control Total	Control Mean	Control SD	MD	95%-CI	Weight (common)	Weight (random)
Iannuzzu et al., 2009	13	73.70	7.3000	13	74.20	7.0000	-0.50	[-6.00; 5.00]	9.5%	10.8%
Kirk et al., 2012	36	61.60	11.4000	31	60.70	10.0200	0.90	[-4.23; 6.03]	10.9%	12.2%
Kong et al., 2013	34	74.30	7.0000	27	72.40	9.8000	1.90	[-2.48; 6.28]	15.0%	16.1%
Ramon-Krauel et al., 2013	7	-7.20	8.2000	9	-5.30	8.4000	-1.90	[-10.09; 6.29]	4.3%	5.1%
Rouhani et al., 2013	19	73.09	4.3600	22	75.89	4.6900	-2.80	[-5.57; -0.03]	37.5%	33.1%
Visuntranukul et al., 2015	25	73.00	7.0000	27	73.00	6.0000	0.00	[-3.56; 3.56]	22.8%	22.7%
**Common effect model**	**134**			**129**			**-0.80**	**[-2.49; 0.90]**	**100.0%**	**--**
**Random effects model**							**-0.66**	**[-2.56; 1.23]**	**--**	**100.0%**
**Prediction interval**								**[-4.35; 3.02]**		

Heterogeneity: $I^2 = 0\%$, $\tau^2 = 0.8269$, $p = 0.53$

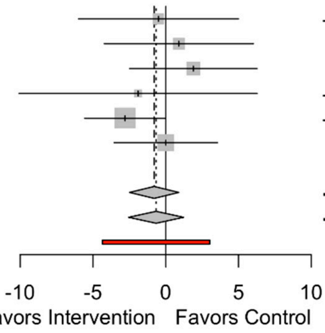

Supplementary Figure S14. Risk of bias assessment presented as traffic lights.

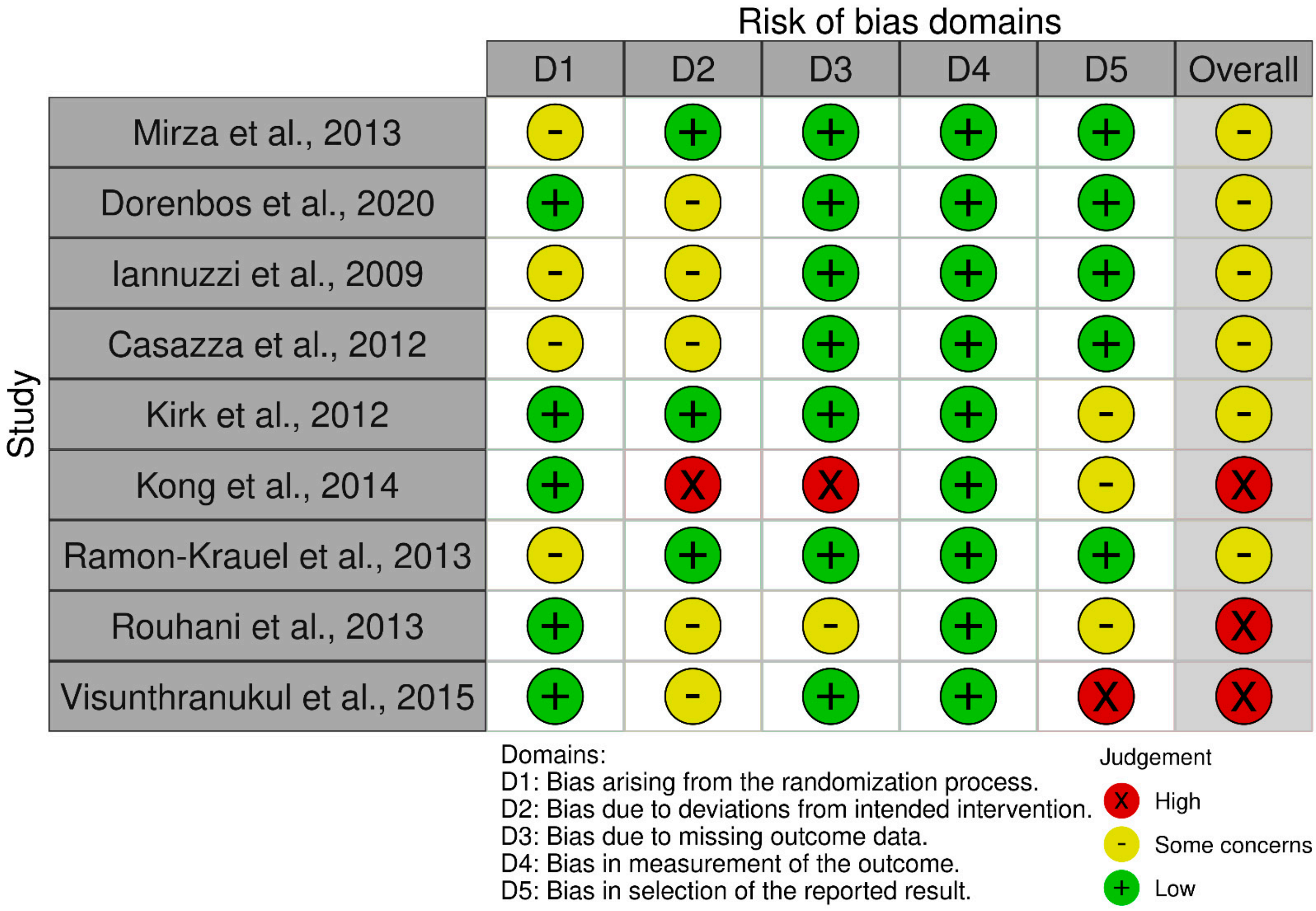

Supplementary Figure S15. Risk of bias assessment presented as bar plots.

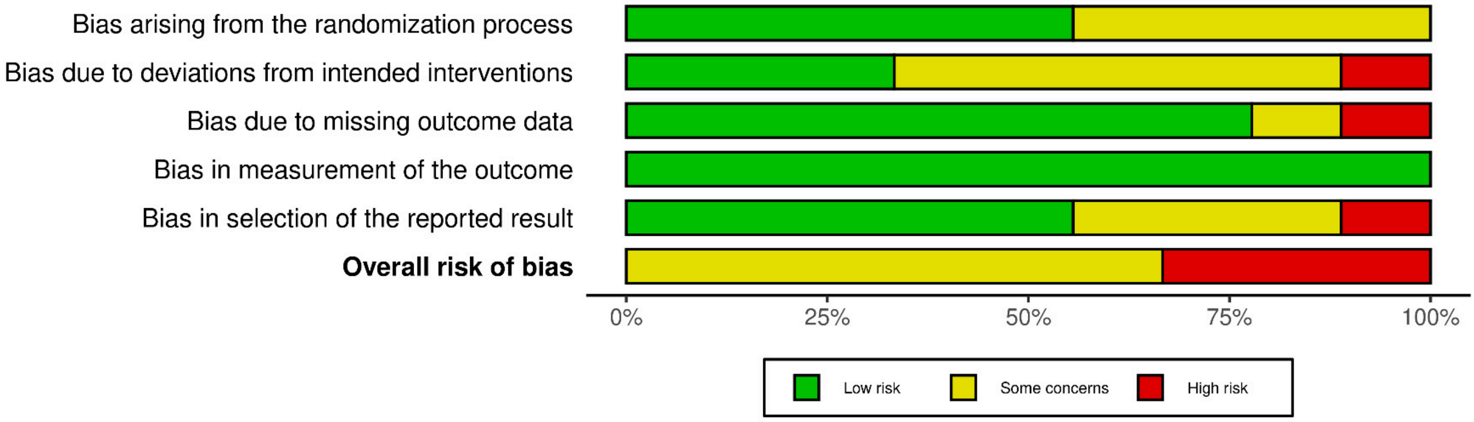

Supplement: Supplementary file 1 [file children-10-01481-s001.zip › children-2585403-supplementary.pdf]
